# Proteomic Analysis of Stationary Growth Stage Adaptation and Nutritional Deficiency Response of *Brucella abortus*

**DOI:** 10.3389/fmicb.2020.598797

**Published:** 2020-12-15

**Authors:** Jianghua Yang, Mengzhi Liu, Jinling Liu, Baoshan Liu, Chuanyu He, Zeliang Chen

**Affiliations:** ^1^Key Laboratory of Livestock Infectious Diseases in Northeast China, Ministry of Education, College of Animal Science and Veterinary Medicine, Shenyang Agricultural University, Shenyang, China; ^2^Tecon Biological Co., Ltd., Urumqi, China; ^3^Beijing Advanced Innovation Center for Soft Matter Science and Engineering, Beijing University of Chemical Technology, Beijing, China; ^4^Brucellosis Prevention and Treatment Engineering Technology Research Center of Inner Mongolia Autonomous Region, Inner Mongolia University for Nationalities, Tongliao, China; ^5^School of Public Health, Sun Yat-sen University, Guangzhou, China

**Keywords:** *Brucella abortus*, proteomics, exponential phase, stationary phase, nutritional stress

## Abstract

Brucellosis, an important bacterial zoonosis caused by *Brucella* species, has drawn increasing attention worldwide. As an intracellular pathogen, the ability of *Brucella* to deal with stress within the host cell is closely related to its virulence. Due to the similarity between the survival pressure on *Brucella* within host cells and that during the stationary phase, a label-free proteomics approach was used to study the adaptive response of *Brucella abortus* in the stationary stage to reveal the possible intracellular adaptation mechanism in this study. A total of 182 downregulated and 140 upregulated proteins were found in the stationary-phase *B. abortus*. *B. abortus* adapted to adverse environmental changes by regulating virulence, reproduction, transcription, translation, stress response, and energy production. In addition, both exponential- and stationary-phase *B. abortus* were treated with short-term starvation. The exponential *B. abortus* restricted cell reproduction and energy utilization and enhanced material transport in response to nutritional stress. Compared with the exponential phase, stationary *Brucella* adjusted their protein expression to a lesser extent under starvation. Therefore, *B. abortus* in the two growth stages significantly differed in the regulation of protein expression in response to the same stress. Overall, we outlined the adaptive mechanisms that *B. abortus* may employ during growth and compared the differences between exponential- and stationary-phase *B. abortus* in response to starvation.

## Introduction

Brucellosis, an important bacterial zoonosis, has drawn increasing attention worldwide. Each year, there are approximately more than 500,000 new cases of brucellosis worldwide, resulting in major losses in animal husbandry. Humans are mainly infected through direct contact with infected animals or the consumption of unpasteurized animal products, such as raw milk (Pappas et al., 2006a; McDermott et al., 2013). Aerosols are also an important transmission route, which makes *Brucella* spp. a potential biological weapon (Pappas et al., 2006b).

Several studies have focused on the virulence factors of *Brucella*, such as lipopolysaccharide (LPS) with atypical lipid A (Cardoso et al., 2006), type IV secretion system (T4SS) VirB (Ke et al., 2015), and VjbR (Ke et al., 2012). However, the pathogenic mechanisms of *Brucella* are not yet well understood. After invasion, *Brucella* sp. stealthily evade the host’s immune system, establish a replication niche inside the host cells, and eventually cause long-term infection (Ahmed et al., 2016). During invasion and the establishment of infection, *Brucella* must overcome a variety of environmental pressures, such as acidity, oxidative stress, and nutritional deficiency (Roop et al., 2009). Remarkably, the survival pressure on *Brucella* within a phagosome is similar to that during the stationary phase (Roop et al., 2003). In the early stage of intracellular infection, the levels of carbon center metabolism, material transport, protein synthesis, and transcription of *Brucella* were decreased at the proteomic level (Lamontagne et al., 2009); of note, similar changes were observed in gram-negative bacteria during the stationary phase (Navarro Llorens et al., 2010). Additionally, inhibition of growth, one of the characteristics of the stationary phase, was also observed early after intracellular infection in *Brucella* (Deghelt et al., 2014). The survival of *Brucella* in the stationary phase and within the host cells is regulated by the same virulence factor Hfq (Robertson and Roop, 1999). The absence of Hfq in *Brucella* leads to impaired stationary-phase physiology and intracellular growth. Therefore, the assessment of changes in protein expression patterns occurring during the switch from the exponential to the stationary phase may assist in the understanding of the pathogenesis of brucellosis. Proteomic techniques have been widely used to compare the protein expression patterns of pathogenic microorganisms under different conditions. Combined with bioinformatics technology, previous studies have annotated, classified, and enriched differentially expressed proteins (DEPs) to screen for potentially important proteins (Al Dahouk et al., 2009; Huang and Chiou, 2011; Al Dahouk et al., 2013; Albrethsen et al., 2013; Zai et al., 2017).

In this study, we investigated the adaptive regulation of *Brucella* during the stationary growth period using a label-free proteomics approach. In addition, *Brucella melitensis* 16 M in the exponential phase was more invasive to epithelial cells than bacteria in the stationary growth phase (Rossetti et al., 2009). Therefore, *Brucella abortus* harvested from exponential and stationary stages were treated with short-term nutritional stress, one of the most common stresses for *Brucella* within host cells. Then, the differences in the response of *B. abortus* to starvation in different growth phases were examined. We aimed to provide important information regarding the intracellular adaptation mechanism of *Brucella* and lay a foundation for further investigation.

## Materials and Methods

### *Brucella* Strains and Experimental Design

Virulent *B. abortus* 2,308 was obtained from Tecon Biological Co., Ltd., (Urumqi, China) and cultured in tryptone soya agar (TSA) or tryptone soya broth (TSB). All experiments related to live *B. abortus* 2,308 were conducted in biosafety level 3 (BSL-3) laboratories. A single colony of *Brucella* was obtained using the streak plate method. The colonies were cultured in TSB medium in a shaking incubator set at 37°C. Bacteria were cultured to exponential or stationary growth stages and then harvested. The GEM medium was used for starvation treatment of *B. abortus*. The components of the GEM medium are as follows: MgSO_4_7H_2_O (0.2 g/L), citric acidH_2_O (2.0 g/L), K_2_HPO_4_ (10.0 g/L), NaNH_4_HPO_4_4H_2_O (3.5 g/L), and glucose (20 g/L; Wang et al., 2019). The cells harvested from the exponential or stationary growth stage were resuspended in the GEM medium and then incubated at 37°C by shaking the incubator for 1.5 h. The pH values of all media in this study were adjusted to 7.0. The exponential- and stationary-phase *B. abortus* cultured separately in TSB medium were called TSBL and TSBS, respectively. The exponential- and stationary-phase *B. abortus* cultures treated with starvation were called GEML and GEMS, respectively. The experimental design and sampling time are shown in Supplementary Figure S1.

### Protein Extraction and Digestion

Cells were harvested, centrifuged (7,000 × *g* at 4°C for 15 min), and then washed three times with sterile phosphate-buffered saline buffer. The bacteria were resuspended in lysis buffer, placed on ice, and lysed on ice through ultrasonic wave breaking. When the lysate became clear, it was centrifuged (40,000 × *g* at 4°C for 30 min). A protein quantification kit based on the bicinchoninic acid method (Thermo Fisher Scientific, Waltham, MA, United States) was used for concentration detection of protein in the supernatant. The cell protein extracts were reduced in 5 mM dithiothreitol at 56°C for 30 min. Then, the reduced cell protein extracts were added to iodoacetamide and incubated at room temperature away from light for 15 min. Finally, trypsin was added at a ratio of 1:50 (trypsin:protein, w/w), and the protein solution was digested overnight at 37°C. Trypsin was added again at a ratio of 1:100 (trypsin:protein, w/w) for a second 4-h digestion. For each condition, three biological replicates were sampled for liquid chromatography-tandem mass spectrometry (LC-MS/MS) analysis.

### High-Performance Liquid Chromatography Fractionation

The tryptic samples were fractionated into fractions by high-pH reverse HPLC with Agilent 300 Extend C18 (5-μm particles, 4.6-mm ID, and 250-mm length). The operation was as follows: peptides were separated with a gradient of 8–32% acetonitrile (pH = 9.0) into 60 fractions over 60 min; then, the peptides were combined into four fractions and dried by vacuum centrifuging for further operation.

### LC-MS/MS Analysis

The tryptic peptides were dissolved in 0.1% (v/v) formic acid (solvent A) and then separated using EASY-nLC 1,200 Ultra High Performance Liquid System. Solvent A contained 0.1% formic acid and 2% acetonitrile, while solvent B contained 0.1% formic acid and 90% acetonitrile. Gradient settings were as follows: 0–30 min, 8–16% solvent B; 30–55 min, 18–32% solvent B; 55–57 min, 32–80% solvent B; and 57–60 min, 80% solvent B. The flow rate was maintained at 400 nl/min.

The peptides were subjected to a nano-electrospray ionization (NSI) source and then analyzed by Orbitrap Fusion^TM^ Lumos mass spectrometry (MS). The electrospray voltage applied was 2.0 kV. The intact peptides were detected in the Orbitrap at a resolution of 60,000. The peptides were selected for tandem mass spectrometry (MS/MS) using normalized collision energy setting as 30, and ion fragments were detected in the Orbitrap at a resolution of 15,000. A data-dependent procedure that alternated between one MS scan followed by 20 MS/MS scans was applied for the top 20 precursor ions above a threshold ion count of 5E4 in the MS survey scan, with 30.0 s dynamic exclusion. Automatic gain control (AGC) was used to prevent overfilling of the ion trap; the 5E4 ions were accumulated for the generation of MS/MS spectra. For MS scans, the *m*/*z* scan range was 350–1,550.

### Database Search

The resulting MS/MS data were processed using MaxQuant search engine (v.1.5.2.8). Tandem mass spectra were searched against the UniProt *B. abortus* (3,023 sequences) database concatenated with the reverse decoy database. Trypsin/P was specified as the cleavage enzyme allowing up to two missing cleavages. The mass tolerance for precursor ions of the first search and main search was set to 20 and 5 ppm, respectively. The mass tolerance for fragment ions was set as 0.02 Da. Carbamidomethyl on cysteine was specified as fixed modification, and oxidation on methionine, N-terminal acetylation of proteins, and deamidation of asparagine were specified as variable modifications. The false discovery rate (FDR) for protein identification and peptides and modification sites were set at 1%.

### Bioinformatic Analysis

Gene Ontology (GO) annotation proteome was derived from the Genebank database^1^. Then, proteins were classified by GO annotation based on three categories: biological process, cellular component, and molecular function. The Kyoto Encyclopedia of Genes and Genomes (KEGG) database was used to annotate the pathways of the identified proteins. The Clusters of Orthologous Groups (COG^2^) system software program was used to determine the functional distribution of identified proteins.

### Real-Time Quantitative RT-PCR

The total RNA of *B. abortus* was extracted using TRIzol (Invitrogen), as recommended by the manufacturer. First, 1 μg of total RNA was reverse transcribed to cDNA using HiScript II Q RT SuperMix (Vazyme Biotech). Then, 1 μl of cDNA was amplified in 10 μl volumes using the ChamQ Universal SYBR qPCR Master Mix kit (Vazyme Biotech) and the following thermocycling program: (1) 95°C for 30 s; (2) 40 cycles at 95°C for 10 s and 60°C for 30 s; and (3) 95°C for 15 s, 60°C for 1 min, and 95°C for 15 s. Real-time quantitative RT-PCR (qRT-PCR) analysis was performed in the Applied Biosystems QuantStudio 3 RT-PCR System. The transcript abundances for the target genes in each sample were assayed three times and normalized to that of 16S rRNA, which served as the internal control. Relative gene expression was calculated using the 2^–Δ Δ *Ct*^ method, and the specific primers used in this study are listed in Supplementary Table S1.

## Results

### Overview of the *B. abortus* Proteome

Protein samples of *Brucella* obtained from different growth stages and treated with or without GEM medium were subjected to label-free proteomic analysis. A total of 1,290,057 secondary spectra were obtained using liquid chromatography with MS/MS analysis, and the available effective spectrum number was 605,083 (46.9%). A total of 32,477 peptide fragments were identified, of which the specific peptide fragments were 32,433. We identified 2,185 proteins, which accounted for about 72.3% of the predicted proteome, and 2,001 of these proteins were quantifiable (Supplementary Figure S2A and Supplementary Table S2). The MS/MS data were tested for quality control (Supplementary Figure S2). The molecular weight of the proteins was negatively correlated with their coverage (Supplementary Figure S2B) because proteins with higher molecular weights could produce more enzymatic peptide segments. To achieve the same coverage, larger proteins were needed to identify more peptide segments. The primary mass error of most spectrograms was less than 10 ppm, which is in accordance with the high-precision characteristics of the Orbitrap mass spectrometer, indicating that the mass accuracy of the mass spectrometer was normal (Supplementary Figure S2C). The length distribution of the peptides identified by MS/MS analysis met the quality control requirements. Most of the peptides were distributed in 7–20 amino acid fragments, which is in accordance with the general rules based on trypsin enzymatic hydrolysis and higher-energy collisional dissociation (Supplementary Figure S2D).

### Differentially Expressed Proteins in Different Groups

*Brucella abortus* grown in TSB medium were cultured to the exponential or stationary growth stage, harvested, and then starved for 1.5 h. Protein samples were subjected to label-free quantitative proteomic analysis. The proteins were divided into the following four comparison groups for analysis: TSBSL (TSBS vs. TSBL), GEMSL (GEMS vs. GEML), GTLL (GEML vs. TSBL), and GTSS (GEMS vs. TSBS). The TSBSL group was employed to analyze the adaptive regulation of *B. abortus* in the stationary phase, whereas the latter three groups were used to study the response of *Brucella* to nutritional stress in different growth stages.

**FIGURE 1:**
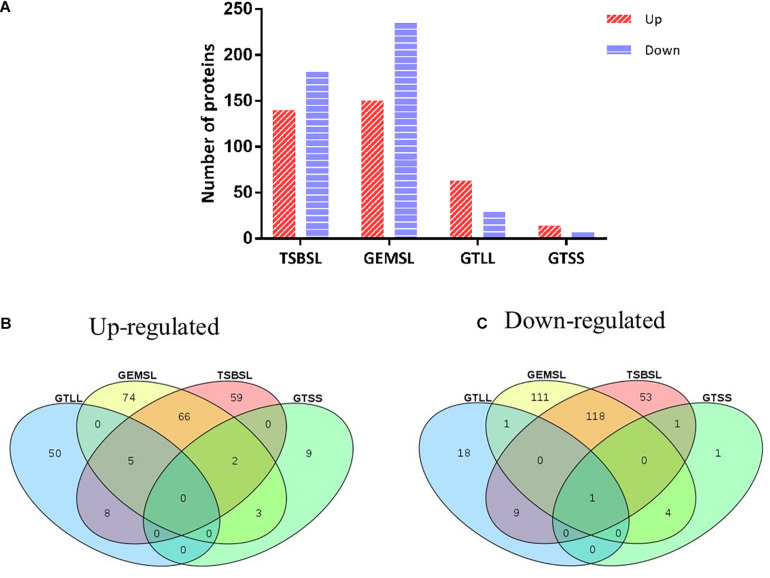
Statistical analysis of differentially expressed proteins (DEPs). **(A)** Number of up- or downregulated proteins in the groups of TSBSL, GEMSL, GTLL, and GTSS; **(B)** a Venn diagram of upregulated proteins in the groups of TSBSL, GEMSL, GTLL, and GTSS; and **(C)** a Venn diagram of downregulated proteins in the groups of TSBSL, GEMSL, GTLL, and GTSS. TSBSL group: stationary-phase *Brucella abortus* versus exponential-phase *B. abortus* cultured in the tryptone soya broth (TSB) medium; GEMSL group: stationary-phase *B. abortus* treated with starvation versus exponential-phase *B. abortus* treated with starvation; GTLL group: exponential-phase *B. abortus* treated with starvation versus untreated exponential-phase *B. abortus*; and GTSS group: stationary-phase *B. abortus* treated with starvation versus untreated stationary-phase *B. abortus*.

Differentially expressed proteins in the four groups were selected using a cutoff of 1.5 fold change and a *p* value of less than 0.05 (Supplementary Table S3). The expression level was upregulated for 140 proteins and downregulated for 182 proteins in the TSBSL group. In the GEMSL group, 150 proteins were more abundant, whereas 235 proteins were less abundant. In the GTLL group, 63 and 29 proteins were upregulated and downregulated, respectively. Lastly, in the GTSS group, 14 proteins were upregulated and seven were downregulated (Figure 1A). Under the same nutritional stress, stationary-phase *B. abortus* induced fewer DEPs than the exponential phase, suggesting that there are differences in the protein regulation patterns in *B. abortus* at different growth stages. In addition, Venn diagrams were constructed to display the relationship between the four comparison groups (Figures 1B,C). In response to nutritional deficiency, one common downregulated protein was found in the two growth stages (Figure 1C) but was not upregulated (Figure 1B). This protein was lipoyl synthase (*lipA*), and lipoate synthase activity may have a significant role in the adaptive regulation of *Brucella*.

### COG Functional Classification of DEPs

**FIGURE 2:**
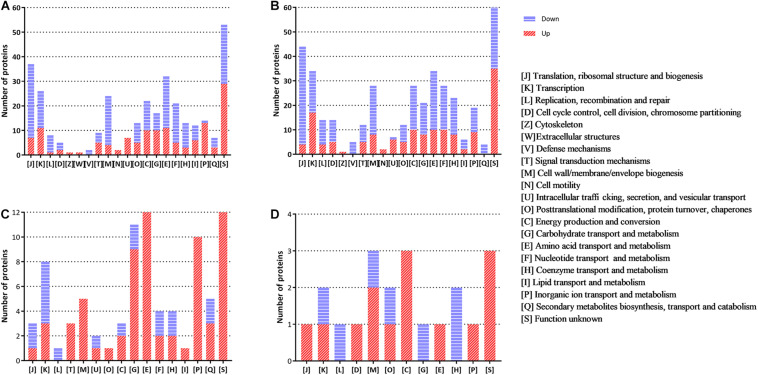
The clusters of orthologous groups (COG) analysis of the differentially expressed proteins for the groups of TSBSL, GEMSL, GTLL, and GTSS. **(A)** COG analysis of differentially expressed proteins (DEPs) for the group of TSBSL; **(B)** COG analysis of DEPs for the group of GEMSL; **(C)** COG analysis of DEPs for the group of GTLL group; and **(D)** COG analysis of DEPs for the group of GTSS group.

The functional distribution of the regulated proteins contributes to the exploration of the adaptation and response of *B. abortus* in the stationary growth phase and under nutritional deficiency. In the stationary phase (Figure 2A), we found that the majority of the downregulated proteins were clustered functionally in the categories of translation, ribosomal structure and biogenesis, transcription, transport, and metabolism of multiple substances as well as cell wall/membrane/envelope biogenesis. These results showed reduced metabolic activity in the stationary phase for *B. abortus*. Seven upregulated DEPs were predicted to be involved in intracellular trafficking, secretion, and vesicular transport, which might suggest that material transport of the stationary-phase *B. abortus* was vigorous. The functional distribution of GEMSL was similar to that of TSBSL (Figure 2B). Under short-term starvation stress, the COG analysis results of DEPs between exponential-phase (Figure 2C) and stationary-phase *B. abortus* (Figure 2D) were different. First, more DEPs were found in the exponential-phase *B. abortus* (Figure 2A). We found that the categories of amino acid as well as inorganic ion transport and metabolism contained 12 and 10 upregulated proteins, respectively, in the stationary-phase *B. abortus* (Figure 2C), whereas only one upregulated protein was found respectively in these categories in the exponential-phase *B. abortus* (Figure 2D). In addition, the transport and metabolism of carbohydrates were different. None of the upregulated proteins were observed in this category in the stationary-phase *B. abortus*, while nine upregulated proteins were found in the exponential-phase *B. abortus*. The COG functional classifications of the four groups are shown in Supplementary Table S4.

### GO Functional Classification of DEPs

The DEPs were annotated using GO functional classification (Supplementary Figure S3). GO annotations can be divided into three major categories: biological process, cellular component, and molecular function. In the biological process category for the TSBSL, GEMSL, and GTLL groups, over 60% of the regulated proteins were related to metabolic and cellular processes. For the GTSS group, the metabolic process was detected in 28.57% of the cases when the cellular process was 25.00%. In terms of cellular component, the majority of the regulated proteins were located in the cell, followed by membrane, protein-containing complex, and organelle. Finally, in the molecular function category, over 80% of the regulated proteins in the four groups were related to catalytic activity and binding, followed by structural molecule activity and transporter activity. The GO functional classifications of the four groups are shown in Supplementary Table S5.

### KEGG Pathway Enrichment Analysis for DEPs

As shown in Table 1, the DEPs in the four groups of TSBSL, GEMSL, GTLL, and GTSS were further investigated using the KEGG database. In the TSBSL group, upregulated proteins were enriched in four pathways, namely ABC transporters, sulfur metabolism, thiamine metabolism, and tuberculosis. The downregulated proteins were enriched in the ribosome. In the GEMSL group, the upregulated proteins were enriched in the two-component system, histidine metabolism, thiamine metabolism, and tuberculosis, and downregulated proteins were enriched in amino sugar and nucleotide sugar metabolism; ribosome, starch, and sucrose metabolism; and galactose metabolism. In the GTLL group, we found two upregulated and downregulated pathways, respectively, including quorum sensing (QS), ABC transporters, histidine metabolism, and the biosynthesis of the siderophore group nonribosomal peptides. In the GTSS group, only one upregulated pathway was observed: glyoxylate and dicarboxylate metabolism.

**TABLE 1:** Kyoto encyclopedia of genes and genomes (KEGG) pathway enrichment analysis for DEPs (Fisher’s exact test, *p* < 0.05).

KEGG Pathway	Pathway Name	Number of Proteins	Fisher’s Exact Test *p* Value
**Upregulated pathway in the TSBSL group**			
map02010	ABC transporters	13	0.021
map00920	Sulfur metabolism	3	0.042
map00730	Thiamine metabolism	3	0.028
map05152	Tuberculosis	2	0.029
**Downregulated pathway in the TSBSL group**			
map03010	Ribosome	19	0.000
**Upregulated pathway in the GEMSL group**			
map02020	Two-component system	8	0.023
map00340	Histidine metabolism	4	0.030
map00730	Thiamine metabolism	4	0.005
map05152	Tuberculosis	2	0.036
**Downregulated pathway in the GEMSL group**			
map00520	Amino sugar and nucleotide sugar metabolism	8	0.046
map03010	Ribosome	23	0.000
map00500	Starch and sucrose metabolism	3	0.043
map00052	Galactose metabolism	5	0.005
**Upregulated pathway in the GTLL group**			
map02010	ABC transporters	11	0.000
map02024	Quorum sensing	8	0.000
**Downregulated pathway in the GTLL group**			
map00340	Histidine metabolism	3	0.001
map01053	Biosynthesis of siderophore group nonribosomal peptides	2	0.001
**Upregulated pathway in the GTSS group**			
map00630	Glyoxylate and dicarboxylate metabolism	2	0.012

### Expression of Virulence-Associated Factors

In this study, the expression levels of some virulence-associated factors were regulated to varying degrees (Table 2). Upregulated virulence factors found in the GEMSL and TSBSL groups were mainly T4SS proteins, transcriptional regulator VjbR (*vjbR*), copper-zinc superoxide dismutase (*sodC*), and integration host factor subunit α (*ihfA*). Decreased expression of the urease subunit α2 (*ureC2*) and accessory protein UreG2 (*ureG2*) was found in the GEMSL and TSBSL groups. Divalent metal cation transporter MntH (*mntH*) was upregulated in the GTLL group, while no regulated virulence factor was found in the GTSS group.

**TABLE 2:** Regulation of classical virulence factors.

Gene Name	Protein Accession	Protein Description	GEMSL Ratio	TSBSL Ratio	GTLL Ratio	GTSS Ratio
***virB4***	Q2YIT8	Type IV secretion system protein virB4	8.74	#N/A	#N/A	#N/A
***virB8***	Q2YJ78	Type IV secretion system protein virB8	24.09	#N/A	#N/A	#N/A
***virB10***	Q2YJ81	Type IV secretion system protein virB10	15.28	23.06	#N/A	#N/A
***virB11***	Q2YJ82	Type IV secretion system protein VirB11	54.03	113.39	#N/A	#N/A
***BAB2_0057***	Q2YJ83	Type IV secretion system putative outer membrane lipoprotein BAB2_0057	#N/A	7.44	#N/A	#N/A
***vjbR***	Q2YJ50	HTH-type quorum sensing-dependent transcriptional regulator VjbR	3.01	3.61	#N/A	#N/A
***sodC***	Q2YKV9	Superoxide dismutase [Cu-Zn]	1.95	2.30	#N/A	#N/A
***ihfA***	Q2YNB8	Integration host factor subunit alpha	3.47	2.60	#N/A	#N/A
***mntH***	Q2YM61	Divalent metal cation transporter MntH	#N/A	3.76	2.70	#N/A
***ureG2***	Q2YQD6	Urease accessory protein UreG 2	0.22	0.09	#N/A	#N/A
***ureC2***	Q2YQD8	Urease subunit alpha 2	0.41	0.45	#N/A	#N/A
***ureE1***	P0C145	Urease accessory protein UreE 1	#N/A	1.84	#N/A	#N/A

*#N/A indicates that the expression level of the protein has no significant difference in the corresponding comparison group.*

### Validation of qRT-PCR

mRNA levels are typically used to infer the corresponding protein abundances (Kuchta et al., 2018). Therefore, to verify the results of proteomics, we selected 10 DEPs and tested their expression at the mRNA level. These DEPs were mainly related to virulence, stress response, material metabolism, and cell division. Most of the results were consistent with those of the proteomic analysis (Table 3); qRT-PCR showed decreased expression of *nrdE*, *ureG2*, *nuoD*, *minD*, and *recA* in the TSBSL and GEMSL groups. The transcription level of *lipA* decreased in the four groups (data in the groups of GTLL and GTSS not shown), and *clpA* decreased in the TSBSL group. However, the changes in transcription of BAB2_0427 and *vjbR* were not consistent with those of protein expression. It is possible that there is a poor linear relationship between mRNA and protein levels.

**TABLE 3:** Transcriptional level of 10 selected genes by RT-qPCR in the groups of TSBSL and GEMSL.

Gene	Protein Accession	Protein Description	TSBSL		GEMSL	
			qPCR	Proteomics	qPCR	Proteomics
***nrdE***	Q2YJZ3	Ribonucleoside-diphosphate reductase	0.072	Down	0.176	Down
***ureG2***	Q2YQD6	Urease accessory protein UreG 2	0.177	Down	0.719	Down
***clpA***	Q2YRJ4	ATP-dependent Clp protease ATP-binding subunit ClpA	0.123	Down	0.420	#N/A
***nuoD***	Q2YNG0	NADH-quinone oxidoreductase subunit D	0.512	Down	0.637	Down
***minD***	Q2YJZ9	Site-determining protein	0.157	Down	0.216	Down
***lipA***	Q2YPV8	Lipoyl synthase	0.348	Down	0.697	Down
***rpoB***	Q2YM15	DNA-directed RNA polymerase subunit beta	0.646	Down	1.006	Down
***recA***	Q2YRU7	Protein RecA	0.135	Down	0.372	Down
***BAB2_0427***	Q2YJ40	ABC transporter, periplasmic substrate-binding protein	0.026	Up	0.140	Up
***vjbR***	Q2YJ50	HTH-type quorum sensing-dependent transcriptional regulator VjbR	0.239	Up	0.489	Up

*#N/A indicates that the expression level of the protein has no significant difference in the corresponding comparison group. The relative transcription levels were determined by the 2^–Δ Δ Ct^ method.*

## Discussion

*Brucella* is an intracellular bacterial pathogen that encounters a broad range of stresses during its life cycle (Roop et al., 2009). The pressures *Brucella* faces within a phagosome are similar to those faced during the stationary phase (Roop et al., 2003). Therefore, studying the proteomic differences of *B. abortus* in the stationary phase may contribute toward a better understanding of its adaptive mechanism within the host cells. Therefore, we analyzed the main protein changes during the transition of *B. abortus* to the stationary phase at the proteomic level. Additionally, to compare the differences between the two growth stages in response to environmental stress, exponential- and stationary-phase *B. abortus* were treated with the same starvation stress and analyzed by the proteomic method. Based on the results of the COG, GO, and KEGG analyses, the DEPs were divided into the following different categories and discussed.

### Regulation of Virulence-Related Factors

T4SS encoded by the *virB* operon (*virB1-12*) plays a crucial role in the establishment of chronic infection in *B. abortus* (Ke et al., 2015). For *B. abortus*, three T4SS proteins were identified as being induced markedly from exponential to stationary phase, especially virB11 (113.4-fold). The persistent establishment of *Brucella* infection depends on its ability to survive and proliferate within the host cells (Byndloss and Tsolis, 2016). After invasion, *Brucella* is contained within a vacuole (BCV), which can control its traffic within the host cell. After a brief interaction between BCV and lysosomes, the undigested *Brucella* reaches the endoplasmic reticulum and establishes the replicative niche (Celli, 2019). T4SS helps BCVs escape from lysosomal killing in host cells (Macedo et al., 2015). In addition, T4SS is required for the biogenesis of the endoplasmic reticulum-derived replication niche through the secretion and translocation of several effector proteins (Marchesini et al., 2011; Myeni et al., 2013). Several proteins participate in T4SS regulation, including LuxR-type regulator VjbR and integration host factor (Sieira et al., 2004; de Jong et al., 2008; Caswell et al., 2012), which showed increased abundance during the stationary phase in this study.

Copper-zinc superoxide dismutase (Cu/ZnSOD) was more abundant in the TSBSL and GEMSL groups. Cu/ZnSOD protects *B. abortus* from oxidative damage, thus supporting intracellular survival and replication (Jacobsen et al., 2005; Pratt et al., 2015). In addition, the divalent metal cation transporter MntH showed an increased expression level in the TSBSL group. MntH is responsible for manganese transport and plays a critical role in the virulence of *B. abortus* 2,308 (Anderson et al., 2009). Surprisingly, two urease-related proteins were repressed during the switch from the exponential to the stationary phase. Urease is a nickel-containing polysubunit enzyme that catalyzes the hydrolysis of urea, resulting in the production of two molecules of ammonia and one molecule of carbon dioxide (Mobley and Hausinger, 1989; Burne and Chen, 2000). Urease enhances the resistance of many pathogenic bacteria to an acidic environment, such as *Helicobacter pylori* (Debowski et al., 2017) and *B. abortus* (Sangari et al., 2007). *Brucella* sp. have two urease operons: *ure1* and *ure2*. The *ure1* operon encoded by *ureDABCEFG* genes is indispensable for urease activity, and *ure2* is composed of *ureABCEFGDT* genes that encode the nickel and urea transport systems (Bandara et al., 2007; Sangari et al., 2007, 2010). The reduction of urease-related proteins could be due to a reduction in the demand for ammonia assimilation. Generally, during the stationary phase of *B. abortus*, the expression levels of some virulence-related factors were induced.

### Cell Division and DNA Replication

In stationary-phase *B. abortus*, the expression levels of some important proteins involved in cell division and DNA replication were downregulated, such as cell division topological specificity factor MinE (*minE*), site-determining protein MinD (*minD*), chromosomal replication initiator protein DnaA (*dnaA*), DNA-directed DNA polymerase (BAB1_0845), and replicative DNA helicase (BAB1_0475). During cell division, the FtsZ ring divides cells equally by forming a diaphragm in the middle of the cell. In gram-negative bacteria, three Min proteins (MinC, MinD, and MinE) prevent the placement of the FtsZ ring in the wrong region (Margolin, 2001; Weiss, 2004). In addition, the expression level of ribonucleoside-diphosphate reductase (*nrdE*) was 20.4-fold downregulated. The enzyme is responsible for catalyzing the biosynthesis of deoxyribonucleotides, which are essential precursors for DNA replication (Reichard, 1993). The decreased expression of these proteins indicates that cell replication of *B. abortus* is inhibited during the stationary stage. Invasive *B. abortus* is mainly in the Gap 1 phase of the cell cycle and maintains a non-proliferative state during the first stage of host cell infection (Deghelt et al., 2014). DNA replication occurring in harsh environments may be detrimental. For example, an acidic pH and reactive oxygen species can lead to DNA damage. Therefore, the inhibition of cell division and DNA replication may be a common strategy for *Brucella* to cope with environmental stress.

Under starvation treatment, DNA replication and the cell division of *B. abortus* in the two growth stages were not significantly affected because a few DEPs were observed in the above two categories. The duration of starvation treatment may be short for *B. abortus*, and the effects on cell division were not significant. Prolonging the starvation treatment time may yield different results.

### Transcription

Two components of RNA polymerase (RNAP) core enzymes, rpoB and rpoC, were repressed in the stationary phase. RNAP core enzymes participate in the synthesis of various RNAs and play an important role in the bacterial transcription process (Sutherland and Murakami, 2018). Three proteins, described as the cold shock DNA-binding domain, encoded by BAB1_1284 and BAB1_1512, respectively, showed reduced expression levels. Some cold shock proteins play a role in RNA chaperone activity and participate in the RNA folding process (Rennella et al., 2017). In addition, the expression levels of some bacterial regulatory proteins related to transcriptional regulation decreased. These findings suggest that *B. abortus* significantly reduced its own transcription level to adapt to environmental changes during the stationary growth period.

For stationary-phase *B. abortus*, two bacterial regulatory proteins were regulated under nutrient restriction. However, for exponential-phase *B. abortus*, more transcription-related DEPs were found according to the COG analysis, including three upregulated and five downregulated proteins. The downregulated proteins were four bacterial regulatory proteins, and one σ factor belonging to the σ^70^ family was involved in transcription initiation (Paget and Helmann, 2003). This result is easy to be accepted because reduction of transcription levels leads to energy saving, which may be crucial for *B. abortus* in response to starvation.

### Protein Synthesis

The expression of 18 ribosomal proteins was downregulated, representing approximately 10% of coverage of all downregulated proteins during the stationary stage. Ribosomes are molecular machines for protein synthesis and are responsible for transforming genetic codes into amino acid sequences (Opron and Burton, 2018). Reduced expression levels occurred for some enzymes involved in the synthesis of various amino acids, such as glutamate, tryptophan, and branched-chain amino acids, suggesting that *B. abortus* reduced their metabolic activity. It is not surprising that the level of protein synthesis showed a decline because the stationary phase does not require large amounts of proteins; these are usually needed for rapid cell division.

Under nutritional stress, the expression levels of one ribosomal protein increased in stationary-phase *B. abortus*. Interestingly, the translational regulation in the starved exponential-phase *B. abortus* was different, and methionine-tRNA ligase (*metG*) showed a decreased abundance, which is a critical enzyme for the translation initiation and elongation of protein synthesis by catalyzing the incorporation of methionine to its transfer RNA (tRNA; Wiltrout et al., 2012). The simultaneous repression of transcription- and translation-related processes may indicate that the exponential-phase *B. abortus* deals with starvation stress by reducing its metabolic activity.

### Cellular Stress Response

This group of proteins is mainly related to DNA repair, protein folding, or oxidative stress. One reduced protein involved in the SOS response, protein RecA (*recA*), was observed during the transition from the exponential to stationary phase. The SOS response is an important regulatory system for bacteria to cope with DNA damage, which is regulated by the promoter protein RecA (Janion, 2008; Žgur-Bertok, 2013). In addition, the abundance of some proteins involved in protein folding also decreased. Chaperonin clpA (*clpA*) can promote specific proteins to unfold and degrade (Singh et al., 2001). The chaperone protein DnaJ (BAB1_2025) as co-chaperone of heat shock protein (Hsp) 70 assists in the folding of nascent proteins, refolding, and degradation of misfolded proteins (Qiu et al., 2006). The reduction of these proteins in the stationary phase indicates that DNA damage and protein misfolding occur more frequently during the exponential phase. In contrast, peroxiredoxin (BAB1_0504), glutaredoxin (*grxC*), and Cu/ZnSOD (*sodC*) were upregulated, and these are required for the detoxification of reactive oxygen species (Miao and St Clair, 2009; Rhee, 2016; Ma et al., 2018). The induced expression of stress-related proteins is not surprising because of the rapid consumption of nutrients and the accumulation of toxic metabolites in the stationary phase.

The overexpression of more stress-related proteins was found in the exponential-phase *B. abortus* in response to starvation. The protein-methionine-sulfoxide reductase catalytic subunit MsrP (*msrP*) and glutathione S-transferase (BAB2_0230) were 1.71- and 3.06-fold upregulated, respectively, and are responsible for maintaining the redox state of cells and protecting cells from oxidative damage (Allocati et al., 2009; Tarrago et al., 2018). For stationary-phase *B. abortus*, there was no upregulated protein closely related to the stress response. Therefore, *B. abortus* in the exponential phase shows a broader response and mobilizes antistress proteins in time to deal with adverse environmental changes compared with stationary-phase *B. abortus*.

### Energy Production and Conversion

During the transition from the exponential to stationary phase, metabolic downshift occurred in *B. abortus*. Two NADH-quinone oxidoreductase subunits encoded by *nuoB* and *nuoD*, respectively, showed decreased expression levels during the stationary growth period. NADH-quinone oxidoreductase plays a pivotal role in energy production (Novakovsky et al., 2016). The limited utilization of energy may be an important strategy for stationary-phase *B. abortus* to adapt to survival pressure. The abundance of holo-[acyl carrier protein] (ACP) synthase (*a*) related to the fatty acid biosynthetic process increased. ACP shuttles acyl intermediates to the fatty acid synthase system. ACP initially exists in the form of inactive apo-ACP and is then modified by holo-ACP synthase to become active (White et al., 2005; Chan and Vogel, 2010; Hiltunen et al., 2010). The increased synthesis of fatty acids is likely for energy storage, which might be conducive to the long-term survival of *B. abortus* under starvation.

Under starvation treatment, decreased expression levels of phosphogluconate dehydratase (*edd*) involved in the Entner–Doudoroff (ED) pathway were observed in the exponential-phase *B. abortu*s. The ED pathway is one of the most common glycolytic routes in bacteria but has an inferior energy efficiency (Klingner et al., 2015). The above results show that the ED pathway might not be the optimum method of acquiring energy under starvation stress. Additionally, some enzymes related to the transport and metabolism of amino acids and carbohydrates were significantly upregulated. This suggests that the exponential-phase *B. abortu*s carries out complex nutrient metabolism and transformation in response to starvation. For the stationary-phase *B. abortus*, glycine cleavage system (GCS) H-protein (*gcvH*) showed an increased abundance in the starved stationary-phase *B. abortus*. The GCS is an important pathway in glycine degradation and is widely distributed in animals, plants, and bacteria (Kikuchi et al., 2008). Increased levels of H-protein may suggest an increased utilization of glycine in the starved stationary-phase *B. abortus*. Of note, it is remarkable that *lipA*, which is responsible for lipoic acid biosynthesis, was downregulated in all groups (Figure 1C). Lipoic acid as a sulfur-containing cofactor is required for the lipoylation of pyruvate dehydrogenase or α-ketoglutarate dehydrogenase in the citric acid cycle. Therefore, this result suggests that energy production may be limited in *B. abortus* under growth phase transition or nutritional restriction.

### Enriched KEGG Pathways

In the stationary phase, one upregulated pathway was ABC transporters in *Brucella*. These transporters are located in the cytoplasmic membrane or periplasmic space and are responsible for the transport and metabolism of carbohydrate, amino acid, and ion (data not shown). Additionally, the upregulation of sulfur and thiamine metabolism was observed, indicating that the utilization of sulfur and thiamine in the stationary-phase *Brucella* increased. The ribosome related pathway was downregulated, consistent with the results concerning protein synthesis in the stationary phase.

For exponential *B. abortus* under starvation, the ABC transporter pathway was also upregulated, suggesting that there was an active substance transport in exponential-phase *Brucella* in response to starvation. Five of the transporters were also involved in the QS pathway, which allows bacteria to adapt to environmental changes as a collective entity (Waters and Bassler, 2005). Meanwhile, the histidine metabolism pathway was potentially downregulated, and the expression of HutI and Huth, two important enzymes in the histidine utilization (Hut) system (Bender, 2012), decreased synchronously. This may be due to the sudden decrease in histidine availability in the medium, which also indicates that histidine is an important carbon or nitrogen source for the growth and metabolism of *Brucella*. Interestingly, fewer pathway changes were observed in *B. abortus* under starvation in the stationary phase, compared with exponential *B. abortus*; only the glyoxylate and dicarboxylate metabolism pathway, crucial for the synthesis of carbohydrates via the conversion of acetyl CoA (Borjian et al., 2017), was upregulated.

Currently, the proteomic changes of intracellular *Brucella* have been comprehensively analyzed. Compared with the published results (Lamontagne et al., 2009), we found that the adaptation of *Brucella* in the stationary phase was similar to that in the early stage of intracellular infection. Most significantly, the expression of proteins (including RNA and protein synthesis-related ones) decreased in *Brucella* under both conditions. Remarkably, the decreased expression of NADH-1 subunits indicated that a low oxygen tension-based respiration was adopted in the stationary phase and early stage of intracellular infection. Moreover, in the early stage of infection, LPS (one of the cell wall components) synthesis-related protein expression decreased. Similarly, during the stationary phase, the expression of peptidoglycan synthesis-related enzymes, such as glycosyltransferase (BAB1_0932 and BAB1_0114), decreased. However, there were some differences with respect to cell division. The division of *Brucella* seems to be inactive in both the early stage of intracellular infection and the stationary phase. However, several proteins related to DNA replication and cell division were upregulated in the early stage of intracellular infection. Overall, our results suggest that in the context of proteomics, the physiological status of *Brucella* in the stationary phase *in vitro* and in the early stage of intracellular infection is indeed similar. Therefore, stationary-phase *Brucella* may be a good model for the study of intracellular adaptation.

Interestingly, *B. abortus* in the two growth stages showed different proteomic changes in response to starvation stress. Compared with the stationary phase, the exponential *B. abortus* induced more DEPs. Protein synthesis and transcription were significantly inhibited when increased expression of oxidative stress-associated proteins was observed in starved exponential-phase *B. abortus*. Additionally, more active substance material transport occurred in exponential-phase *B. abortus*. A previous published study indicated that *B. melitensis* at the exponential phase were more invasive to host cells than at the stationary growth phase (Rossetti et al., 2009). Our results suggest that exponential-phase *B. abortus* can make timely metabolic adjustments in response to the challenge of starvation, which contributes to the high invasiveness of exponential-phase *Brucella*.

## Conclusion

To summarize, we explored the proteomic changes in exponential- and stationary-phase *B. abortus*, employing a label-free quantitative proteomics approach. *B. abortus* actively adapted to environmental changes in the stationary phase by regulating virulence, reproduction, transcription, translation, stress response, and energy production. Additionally, the protein expression profiles of exponential- and stationary-phase *B. abortus*, treated with nutrient restriction, were compared and analyzed. First, more DEPs were identified in exponential-phase *B. abortus*, including 63 upregulated and 29 downregulated proteins. Exponential-phase *B. abortus* attempt to restrict metabolic activity, resulting in energy savings. Overall, based on the proteomic data, several insights are provided for the intracellular adaptation mechanism of *Brucella*. In the future, efforts will focus on some of the important DEPs identified in this study.

## Data Availability Statement

The datasets generated in this study can be found in online repositories. The names of the repository/repositories and accession number(s) can be found below: https://www.ebi.ac.uk/pride/archive/, PXD020869.

## Author Contributions

ZC designed the experiments. ML prepared protein samples for LC-MS/MS analysis. ZC, BL, CH, and JL carried out the revision of manuscript. JY analyzed the data and drafted the manuscript. All authors contributed to the article and approved the submitted version.

## Conflict of Interest

ML was employed by the company of Tecon Biological Co., Ltd. The remaining authors declare that the research was conducted in the absence of any commercial or financial relationships that could be construed as a potential conflict of interest.

## Footnotes

1 https://cipotato.org/genebankcip/

2 http://www.ncbi.nlm.nih.gov/COG/

## Supplementary Material

Supplementary Figure 1 Experimental design of the quantitative proteomics. A label-free proteomics approach was used to study the stationary growth stage adaption and nutritional deficiency response of *Brucella abortus*. Protein samples were collected from the exponential (letter a) and stationary phases (letter b) and then treated with a short-term starvation stress. The identified proteins were analyzed using the GO, COG and KEGG databases.

Supplementary Figure 2 Coverage of the *Brucella abortus* proteome and quality control testing of mass spectrometry.**(A)** Coverage of the *B. abortus* proteome. The coverage of proteins identified and quantitated in this work was 72.3% and 66.2%, respectively; **(B)** Protein mass and coverage distribution; **(C)** Peptide mass tolerance distribution; **(D)** Identified peptide length distribution.

Supplementary Figure 3 GO analysis of DEPs for the groups of TSBSL, GEMSL, GTLL, and GTSS. **(A)** GO analysis of DEPs for the group of TSBSL; **(B)** GO analysis of DEPs for the group of GEMSL; **(C)** GO analysis of DEPs for the group of GTLL group; and **(D)** GO analysis of DEPs for the group of GTSS group.

## References

[B1] Ahmed W. Zheng K. Liu Z. F. (2016). Establishment of chronic infection: *Brucella*’s stealth strategy. *Front. Cell. Infect. Microbiol.* 6:30. 10.3389/Fcimb.2016.00030 27014640 PMC4791395

[B2] Al Dahouk S. Jubier-Maurin V. Neubauer H. Köhler S. (2013). Quantitative analysis of the *Brucella suis* proteome reveals metabolic adaptation to long-term nutrient starvation. *BMC Microbiol.* 13:199. 10.1186/1471-2180-13-199 24007556 PMC3844638

[B3] Al Dahouk S. Loisel-Meyer S. Scholz H. C. Tomaso H. Kersten M. Harder A. (2009). Proteomic analysis of *Brucella suis* under oxygen deficiency reveals flexibility in adaptive expression of various pathways. *Proteomics* 9 3011–3021. 10.1002/Pmic.200800266 19526545

[B4] Albrethsen J. Agner J. Piersma S. R. Højrup P. Pham T. V. Weldingh K. (2013). Proteomic profiling of *Mycobacterium tuberculosis* identifies nutrient-starvation-responsive toxin-antitoxin systems. *Mol. Cell Proteomics* 12 1180–1191. 10.1074/Mcp.M112.018846 23345537 PMC3650330

[B5] Allocati N. Federici L. Masulli M. Di Ilio C. (2009). Glutathione transferases in bacteria. *FEBS J.* 276 58–75. 10.1111/J.1742-4658.2008.06743.X 19016852

[B6] Anderson E. S. Paulley J. T. Gaines J. M. Valderas M. W. Martin D. W. Menscher E. (2009). The manganese transporter Mnth is a critical virulence determinant for *Brucella abortus* 2308 in experimentally infected mice. *Infect. Immun.* 77 3466–3474. 10.1128/Iai.00444-09 19487482 PMC2715675

[B7] Bandara A. B. Contreras A. Contreras-Rodriguez A. Martins A. M. Dobrean V. Poff-Reichow S. (2007). *Brucella suis* urease encoded by Ure1 but not Ure2 is necessary for intestinal infection of Balb/C mice. *BMC Microbiol.* 7:57. 10.1186/1471-2180-7-57 17578575 PMC1983905

[B8] Bender R. A. (2012). Regulation of the histidine utilization (Hut) system in bacteria. *Microbiol. Mol. Biol. Rev.* 76 565–584. 10.1128/Mmbr.00014-12 22933560 PMC3429618

[B9] Borjian F. Johnsen U. Schönheit P. Berg I. A. (2017). Succinyl-coa:mesaconate coa-transferase and Mesaconyl-Coa hydratase, enzymes of the methylaspartate cycle in haloarcula hispanica. *Front. Microbiol.* 8:1683. 10.3389/Fmicb.2017.01683 28932214 PMC5592240

[B10] Burne R. A. Chen Y. Y. (2000). Bacterial ureases in infectious diseases. *Microbes Infect.* 2 533–542. 10.1016/S1286-4579(00)00312-9 10865198

[B11] Byndloss M. X. Tsolis R. M. (2016). *Brucella* Spp. virulence factors and immunity. *Annu. Rev. Anim. Biosci.* 4 111–127. 10.1146/Annurev-Animal-021815-111326 26734887

[B12] Cardoso P. G. Macedo G. C. Azevedo V. Oliveira S. C. (2006). *Brucella* spp noncanonical lps: structure, biosynthesis, and interaction with host immune system. *Microb. Cell Fact.* 5:13. 10.1186/1475-2859-5-13 16556309 PMC1435926

[B13] Caswell C. C. Gaines J. M. Roop R. M. II (2012). The RNA chaperone Hfq independently coordinates expression of the virb type Iv secretion system and the Luxr-type regulator babr in *Brucella abortus* 2308. *J. Bacteriol.* 194 3–14. 10.1128/Jb.05623-11 22020650 PMC3256608

[B14] Celli J. (2019). The intracellular life cycle of *Brucella* Spp. *Microbiol. Spectr.* 7:6. 10.1128/Microbiolspec.Bai-0006-2019 30848234 PMC6448592

[B15] Chan D. I. Vogel H. J. (2010). Current understanding of fatty acid biosynthesis and the acyl carrier protein. *Biochem. J.* 430 1–19. 10.1042/Bj20100462 20662770

[B16] de Jong M. F. Sun Y. H. Den Hartigh A. B. Van Dijl J. M. Tsolis R. M. (2008). Identification of Vcea and Vcec, two members of the Vjbr regulon that are translocated into macrophages by the *Brucella* type Iv secretion system. *Mol. Microbiol.* 70 1378–1396. 10.1111/J.1365-2958.2008.06487.X 19019140 PMC2993879

[B17] Debowski A. W. Walton S. M. Chua E. G. Tay A. C. Liao T. Lamichhane B. (2017). *Helicobacter* pylori gene silencing in vivo demonstrates urease is essential for chronic infection. *PLoS Pathog.* 13:E1006464. 10.1371/Journal.Ppat.1006464 28644872 PMC5500380

[B18] Deghelt M. Mullier C. Sternon J. F. Francis N. Laloux G. Dotreppe D. (2014). G1-arrested newborn cells are the predominant infectious form of the pathogen *Brucella abortus*. *Nat. Commun.* 5:4366. 10.1038/Ncomms5366 25006695 PMC4104442

[B19] Hiltunen J. K. Chen Z. Haapalainen A. M. Wierenga R. K. Kastaniotis A. J. (2010). Mitochondrial fatty acid synthesis–an adopted set of enzymes making a pathway of major importance for the cellular metabolism. *Prog. Lipid Res.* 49 27–45. 10.1016/J.Plipres.2009.08.001 19686777

[B20] Huang C. H. Chiou S. H. (2011). Proteomic analysis of upregulated proteins in *Helicobacter* pylori under oxidative stress induced by hydrogen peroxide. *Kaohsiung J. Med. Sci.* 27 544–553. 10.1016/J.Kjms.2011.06.019 22208537 PMC11916125

[B21] Jacobsen I. Gerstenberger J. Gruber A. D. Bossé J. T. Langford P. R. Hennig-Pauka I. (2005). Deletion of the ferric uptake regulator fur impairs the in vitro growth and virulence of *Actinobacillus pleuropneumoniae*. *Infect. Immun.* 73 3740–3744. 10.1128/Iai.73.6.3740-3744.2005 15908404 PMC1111875

[B22] Janion C. (2008). Inducible sos response system of dna repair and mutagenesis in *Escherichia Coli*. *Int. J. Biol. Sci.* 4 338–344. 10.7150/Ijbs.4.338 18825275 PMC2556049

[B23] Ke Y. Wang Y. Li W. Chen Z. (2015). Type iv secretion system of *Brucella* Spp. and its effectors. *Front. Cell. Infect. Microbiol.* 5:72. 10.3389/Fcimb.2015.00072 26528442 PMC4602199

[B24] Ke Y. Wang Y. Yuan X. Zhong Z. Qu Q. Zhou D. (2012). Altered transcriptome Of The *B. melitensis* vaccine candidate 16mδvjbr, implications for development of genetically marked live vaccine. *Indian J. Microbiol.* 52 575–580. 10.1007/S12088-012-0293-8 24293713 PMC3516643

[B25] Kikuchi G. Motokawa Y. Yoshida T. Hiraga K. (2008). Glycine cleavage system: reaction mechanism, physiological significance, and hyperglycinemia. *Proc. Jpn. Acad. Ser. B Phys. Biol. Sci.* 84 246–263. 10.2183/Pjab.84.246 18941301 PMC3666648

[B26] Klingner A. Bartsch A. Dogs M. Wagner-Döbler I. Jahn D. Simon M. (2015). Large-scale 13c flux profiling reveals conservation of the entner-doudoroff pathway as a glycolytic strategy among marine bacteria that use glucose. *Appl. Environ. Microbiol.* 81 2408–2422. 10.1128/Aem.03157-14 25616803 PMC4357956

[B27] Kuchta K. Towpik J. Biernacka A. Kutner J. Kudlicki A. Ginalski K. (2018). Predicting proteome dynamics using gene expression data. *Sci. Rep.* 8:13866. 10.1038/S41598-018-31752-4 30217992 PMC6138643

[B28] Lamontagne J. Forest A. Marazzo E. Denis F. Butler H. Michaud J. F. (2009). Intracellular adaptation of *Brucella abortus*. *J. Proteome Res.* 8 1594–1609. 10.1021/Pr800978p 19216536 PMC2771391

[B29] Ma H. Wang M. Gai Y. Fu H. Zhang B. Ruan R. (2018). Thioredoxin and glutaredoxin systems required for oxidative stress resistance, fungicide sensitivity, and virulence of alternaria alternata. *Appl. Environ. Microbiol.* 84:e00086-18. 10.1128/Aem.00086-18 29752269 PMC6029089

[B30] Macedo A. A. Silva A. P. Mol J. P. Costa L. F. Garcia L. N. Araújo M. S. (2015). The abcedcba-encoded abc transporter and the virb operon-encoded type IV secretion system of *Brucella ovis* are critical for intracellular trafficking and survival in ovine monocyte-derived macrophages. *PLoS One* 10:E0138131. 10.1371/Journal.Pone.0138131 26366863 PMC4569489

[B31] Marchesini M. I. Herrmann C. K. Salcedo S. P. Gorvel J. P. Comerci D. J. (2011). In search of *Brucella abortus* type IV secretion substrates: screening and identification of four proteins translocated into host cells through Virb system. *Cell. Microbiol.* 13 1261–1274. 10.1111/J.1462-5822.2011.01618.X 21707904 PMC3139020

[B32] Margolin W. (2001). Bacterial cell division: a moving mine sweeper boggles the mind. *Curr. Biol.* 11 R395–R398. 10.1016/S0960-9822(01)00217-2 11378404

[B33] McDermott J. Grace D. Zinsstag J. (2013). Economics of *Brucellosis* impact and control in low-income countries. *Rev. Off. Int. Epizoot.* 32 249–261. 10.20506/Rst.32.1.2197 23837382

[B34] Miao L. St Clair D. K. (2009). Regulation of superoxide dismutase genes: implications in disease. *Free Radic. Biol. Med.* 47 344–356. 10.1016/J.Freeradbiomed.2009.05.018 19477268 PMC2731574

[B35] Mobley H. L. Hausinger R. P. (1989). Microbial ureases: significance, regulation, and molecular characterization. *Microbiol. Rev.* 53 85–108. 2651866 10.1128/mr.53.1.85-108.1989 PMC372718

[B36] Myeni S. Child R. Ng T. W. Kupko J. J. III Wehrly T. D. Porcella S. F. (2013). *Brucella* modulates secretory trafficking via multiple type IV secretion effector proteins. *PLoS Pathog.* 9:E1003556. 10.1371/Journal.Ppat.1003556 23950720 PMC3738490

[B37] Navarro Llorens J. M. Tormo A. Martínez-García E. (2010). Stationary phase in gram-negative bacteria. *FEMS Microbiol. Rev.* 34 476–495. 10.1111/J.1574-6976.2010.00213.X 20236330

[B38] Novakovsky G. E. Dibrova D. V. Mulkidjanian A. Y. (2016). Phylogenomic analysis of type 1 nadh:quinone oxidoreductase. *Biochem. Mosc.* 81 770–784. 10.1134/S0006297916070142 27449624

[B39] Opron K. Burton Z. F. (2018). Ribosome structure, function, and early evolution. *Int. J. Mol. Sci.* 20:40. 10.3390/Ijms20010040 30583477 PMC6337491

[B40] Paget M. S. Helmann J. D. (2003). The Sigma70 family of sigma factors. *Genome Biol.* 4:203. 10.1186/Gb-2003-4-1-203 12540296 PMC151288

[B41] Pappas G. Papadimitriou P. Akritidis N. Christou L. Tsianos E. V. (2006a). The new global map of human *Brucellosis*. *Lancet Infect. Dis.* 6 91–99. 10.1016/S1473-3099(06)70382-6 16439329

[B42] Pappas G. Panagopoulou P. Christou L. Akritidis N. (2006b). *Brucella* as a biological weapon. *Cell. Mol. Life Sci.* 63 2229–2236. 10.1007/S00018-006-6311-4 16964579 PMC11136069

[B43] Pratt A. J. Didonato M. Shin D. S. Cabelli D. E. Bruns C. K. Belzer C. A. (2015). Structural, functional, and immunogenic insights on Cu, Zn superoxide dismutase pathogenic virulence factors from *Neisseria meningitidis* and *Brucella abortus*. *J. Bacteriol.* 197 3834–3847. 10.1128/Jb.00343-15 26459556 PMC4652047

[B44] Qiu X. B. Shao Y. M. Miao S. Wang L. (2006). The diversity of the Dnaj/Hsp40 family, the crucial partners for Hsp70 chaperones. *Cell. Mol. Life Sci.* 63 2560–2570. 10.1007/S00018-006-6192-6 16952052 PMC11136209

[B45] Reichard P. (1993). From RNA To DNA, why so many ribonucleotide reductases. *Science* 260 1773–1777. 10.1126/Science.8511586 8511586

[B46] Rennella E. Sára T. Juen M. Wunderlich C. Imbert L. Solyom Z. (2017). RNA binding and chaperone activity of the *E. coli* cold-shock protein CSPA. *Nucleic Acids Res.* 45 4255–4268. 10.1093/Nar/Gkx044 28126922 PMC5397153

[B47] Rhee S. G. (2016). Overview on peroxiredoxin. *Mol. Cells* 39 1–5. 10.14348/Molcells.2016.2368 26831451 PMC4749868

[B48] Robertson G. T. Roop R. M. Jr. (1999). The *Brucella abortus* host factor I (Hf-I) protein contributes to stress resistance during stationary phase and is a major determinant of virulence in mice. *Mol. Microbiol.* 34 690–700. 10.1046/J.1365-2958.1999.01629.X 10564509

[B49] Roop R. M. II Gaines J. M. Anderson E. S. Caswell C. C. Martin D. W. (2009). Survival of the fittest: how *Brucella* strains adapt to their intracellular niche in the host. *Med. Microbiol. Immunol.* 198 221–238. 10.1007/S00430-009-0123-8 19830453 PMC3814008

[B50] Roop R. M. II Gee J. M. Robertson G. T. Richardson J. M. Ng W. L. Winkler M. E. (2003). *Brucella* stationary-phase gene expression and virulence. *Annu. Rev. Microbiol.* 57 57–76. 10.1146/Annurev.Micro.57.030502.090803 12730323

[B51] Rossetti C. A. Galindo C. L. Lawhon S. D. Garner H. R. Adams L. G. (2009). *Brucella melitensis* global gene expression study provides novel information on growth phase-specific gene regulation with potential insights for understanding *Brucella*:host initial interactions. *BMC Microbiol.* 9:81. 10.1186/1471-2180-9-81 19419566 PMC2684542

[B52] Sangari F. J. Cayón A. M. Seoane A. García-Lobo J. M. (2010). *Brucella abortus* Ure2 region contains an acid-activated urea transporter and a nickel transport system. *BMC Microbiol.* 10:107. 10.1186/1471-2180-10-107 20380737 PMC2868824

[B53] Sangari F. J. Seoane A. Rodríguez M. C. Agüero J. García Lobo J. M. (2007). Characterization of the urease operon of *Brucella abortus* and assessment of its role in virulence of the bacterium. *Infect. Immun.* 75 774–780. 10.1128/Iai.01244-06 17101645 PMC1828483

[B54] Sieira R. Comerci D. J. Pietrasanta L. I. Ugalde R. A. (2004). Integration host factor is involved in transcriptional regulation of the *Brucella abortus* virb operon. *Mol. Microbiol.* 54 808–822. 10.1111/J.1365-2958.2004.04316.X 15491369

[B55] Singh S. K. Rozycki J. Ortega J. Ishikawa T. Lo J. Steven A. C. (2001). Functional domains of the Clpa and Clpx molecular chaperones identified by limited proteolysis and deletion analysis. *J. Biol. Chem.* 276 29420–29429. 10.1074/Jbc.M103489200 11346657

[B56] Sutherland C. Murakami K. S. (2018). An introduction to the structure and function of the catalytic core enzyme of *Escherichia Coli* RNA polymerase. *Ecosal Plus* 8:e0004-2018. 10.1128/Ecosalplus.Esp-0004-2018 30109846 PMC6095464

[B57] Tarrago L. Grosse S. Siponen M. I. Lemaire D. Alonso B. Miotello G. (2018). Rhodobactersphaeroides methionine sulfoxide reductase P reduces R- and S-diastereomers of methionine sulfoxide from a broad-spectrum of protein substrates. *Biochem. J.* 475 3779–3795. 10.1042/Bcj20180706 30389844

[B58] Wang Y. Ke Y. Duan C. Ma X. Hao Q. Song L. (2019). A small non-coding RNA facilitates *Brucella melitensis* intracellular survival by regulating the expression of virulence factor. *Int. J. Med. Microbiol.* 309 225–231. 10.1016/J.Ijmm.2019.04.002 31054808

[B59] Waters C. M. Bassler B. L. (2005). Quorum sensing: cell-to-cell communication in bacteria. *Annu. Rev. Cell Dev. Biol.* 21 319–346. 10.1146/Annurev.Cellbio.21.012704.131001 16212498

[B60] Weiss D. S. (2004). Bacterial cell division and the septal ring. *Mol. Microbiol.* 54 588–597. 10.1111/J.1365-2958.2004.04283.X 15491352

[B61] White S. W. Zheng J. Zhang Y. M. Rock (2005). The structural biology of type ii fatty acid biosynthesis. *Annu. Rev. Biochem.* 74 791–831. 10.1146/Annurev.Biochem.74.082803.133524 15952903

[B62] Wiltrout E. Goodenbour J. M. Fréchin M. Pan T. (2012). Misacylation Of TRNA with methionine in *Saccharomyces cerevisiae*. *Nucleic Acids Res.* 40 10494–10506. 10.1093/Nar/Gks805 22941646 PMC3488245

[B63] Zai X. Yang Q. Yin Y. Li R. Qian M. Zhao T. (2017). Relative quantitative proteomic analysis of *Brucella abortus* reveals metabolic adaptation to multiple environmental stresses. *Front. Microbiol.* 8:2347. 10.3389/Fmicb.2017.02347 29238329 PMC5712581

[B64] Žgur-Bertok D. (2013). DNA damage repair and bacterial pathogens. *PLoS Pathog.* 9:E1003711. 10.1371/Journal.Ppat.1003711 24244154 PMC3820712

