# Supplementary figures and images for: Proteomic Analysis of Stationary Growth Stage Adaptation and Nutritional Deficiency Response of Brucella abortus
Source: Front Microbiol. 2020 Dec 15;11:598797. doi: 10.3389/fmicb.2020.598797 (PMC7769873; doi:10.3389/fmicb.2020.598797)

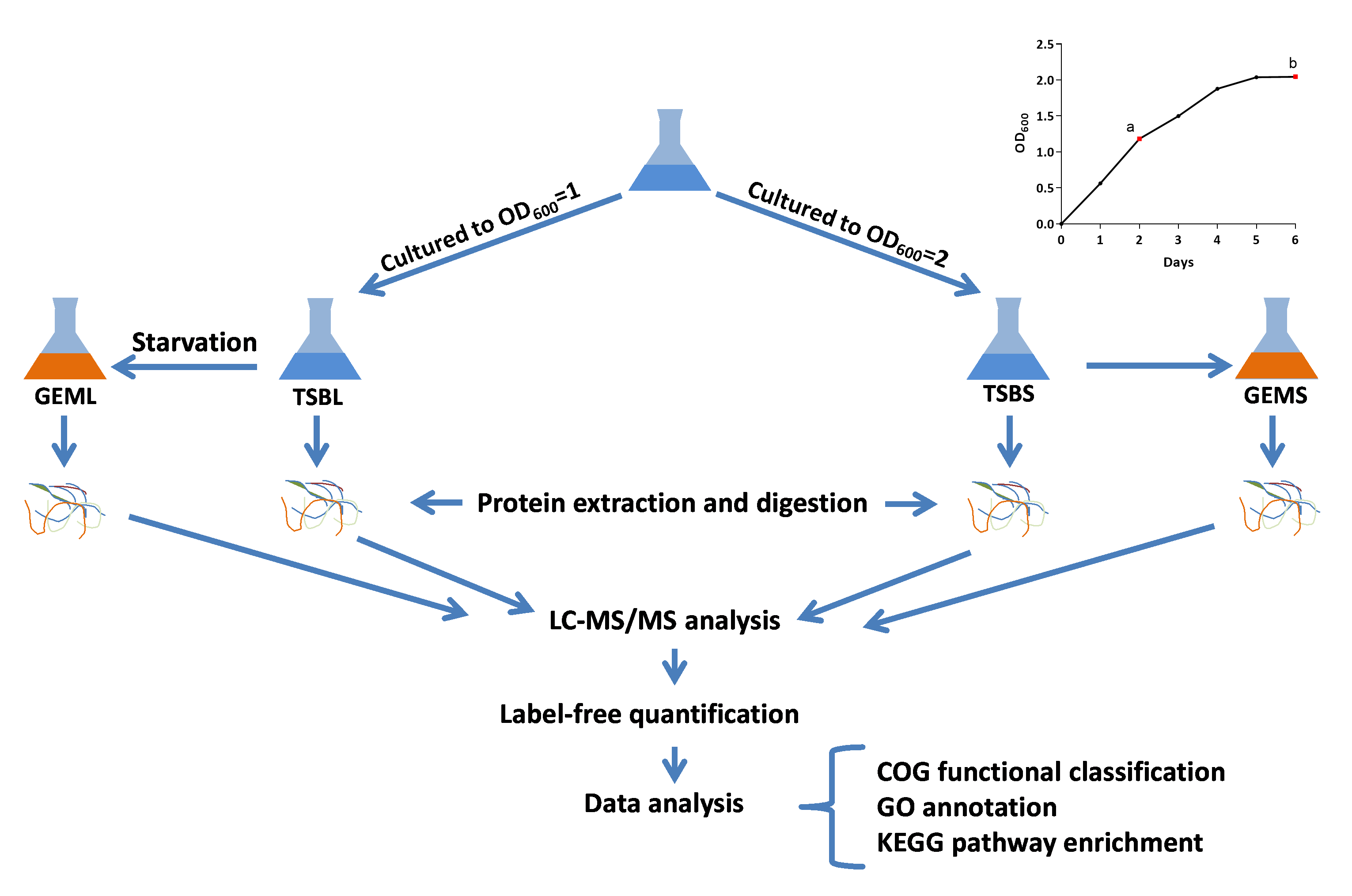

Supplement: Supplementary Figure 1 — Experimental design of the quantitative proteomics. A label-free proteomics approach was used to study the stationary growth stage adaption and nutritional deficiency response of Brucella abortus. Protein samples were collected from the exponential (letter a) and stationary phases (letter b) and then treated with a short-term starvation stress. The identified proteins were analyzed using the GO, COG and KEGG databases. [file Image_1.TIF]

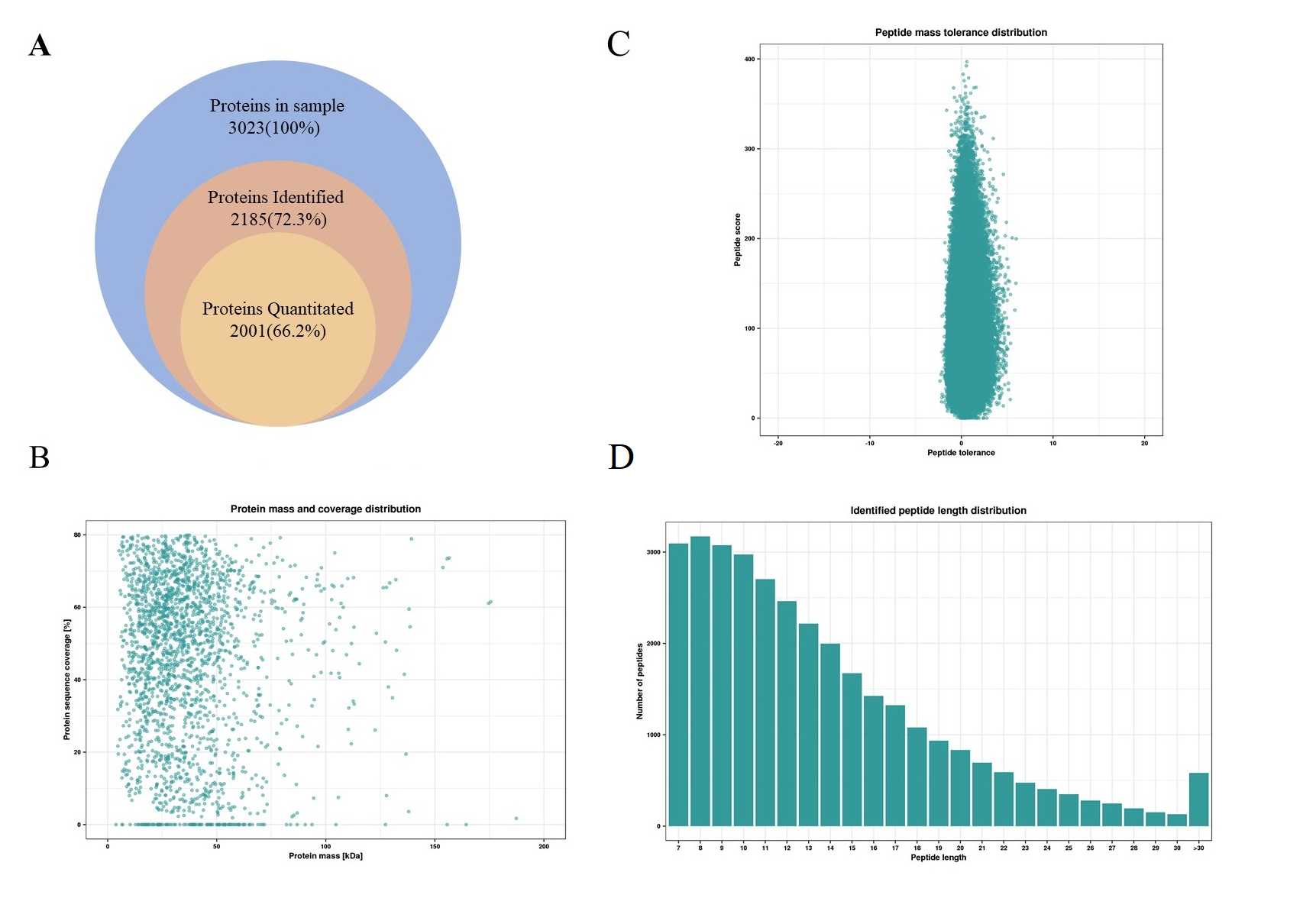

Supplement: Supplementary Figure 2 — Coverage of the Brucella abortus proteome and quality control testing of mass spectrometry.(A) Coverage of the B. abortus proteome. The coverage of proteins identified and quantitated in this work was 72.3% and 66.2%, respectively; (B) Protein mass and coverage distribution; (C) Peptide mass tolerance distribution; (D) Identified peptide length distribution. [file Image_2.TIF]

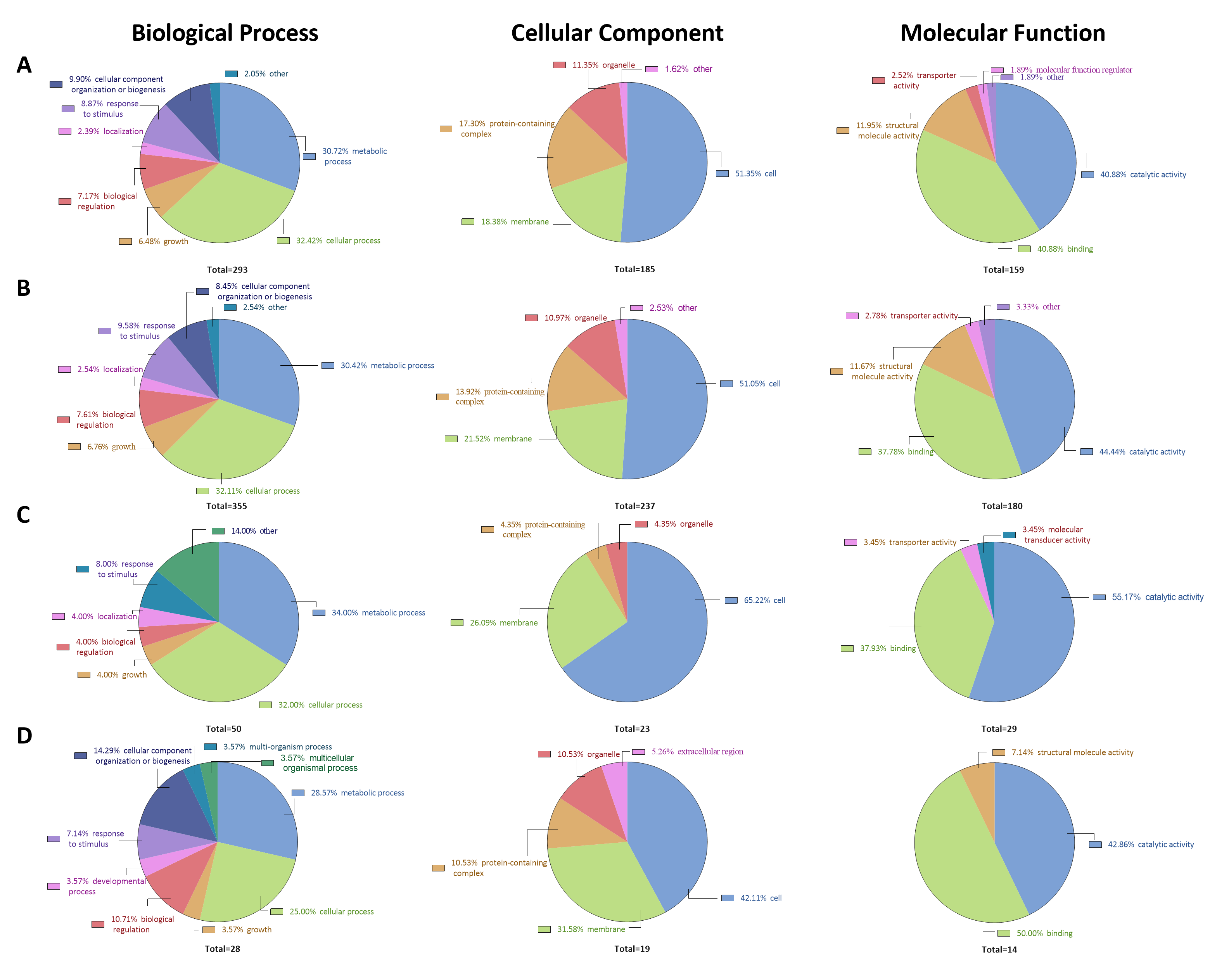

Supplement: Supplementary Figure 3 — GO analysis of DEPs for the groups of TSBSL, GEMSL, GTLL, and GTSS. (A) GO analysis of DEPs for the group of TSBSL; (B) GO analysis of DEPs for the group of GEMSL; (C) GO analysis of DEPs for the group of GTLL group; and (D) GO analysis of DEPs for the group of GTSS group. [file Image_3.TIF]
